# Developing integrative practice on basic soccer skills to stimulate cognitive promotion for children and adolescents

**DOI:** 10.3389/fpsyg.2024.1348006

**Published:** 2024-04-19

**Authors:** Fan Mao, Zelong Li, Chen Qiu, Qun Fang

**Affiliations:** ^1^School of Physical Education, Qingdao University, Qingdao, China; ^2^Research Center for Youth Football, Qingdao University, Qingdao, China

**Keywords:** soccer, cognition, integrative training, dribbling drills, physical education

## Introduction

Soccer is a popular, accessible, and easy-to-organize sport that offers participants opportunities for enjoyable physical activity (Trajković et al., 2020). The characteristics of intermittent running at various levels of intensity associated with jumps, sprints, accelerations, and changes of direction in soccer play benefit physical health and fitness. Due to its positive role in promoting exercise and health behaviors, soccer has been implemented as school-based programs to engage children and adolescents in moderate-to-vigorous physical activity during school hours (Fuller et al., 2010; Barriguete Melendez et al., 2014). An eight-month soccer program induced greater improvement in aerobic fitness and explosive power than standard physical education (PE) protocols for high-school students (Trajković et al., 2020). Consistent findings identified enhanced musculoskeletal fitness of children at 10-12 years of age. Specifically, a greater increase in leg bone mineral density and a larger decline in body fat percentage were identified in the soccer intervention group compared with the control group (Larsen et al., 2023).

In addition to the fitness benefits of school-based soccer programs, influence of soccer play on cognitive development has raised an increasing interest among researchers and educators. As a multi-cognitive sport that integrates decision-making, perception, observation, and action, soccer demands complex cognitive processing involving teammates, opponents, ball, and constantly changing environment (Hicheur et al., 2017). The cognitively challenging nature makes soccer more than a sport for fun or health promotion. Cognitive capabilities are highly plastic during childhood and adolescence (Gogtay et al., 2004). Soccer play provides children and adolescents with affordances to stimulate cognitive development.

The current opinion article proposed that integrating cognitive elements into basic soccer drills induces concurrent benefits in physical fitness and cognitive performance of children and adolescents, thus substantiating integrative practice in the context of school-based soccer. To validate this opinion, an initial examination focused on evidence for the enhanced cognitive performance associated with soccer play. Further discussions offer examples of integrating cognitive elements into basic drills. Following the examples that highlight the idea of integrative training, PE teachers can design mentally challenging soccer drills to stimulate motor and cognitive development (Kolovelonis et al., 2022).

## Influence of soccer play on cognitive performance

Research based on cross-sectional design identified superior cognitive performance of soccer players over non-athletes in executive control, attention, working memory, vigilance, and cognitive flexibility (Vestberg et al., 2012; Ballester et al., 2015; Verburgh et al., 2016; Moratal et al., 2020). In the comparisons between soccer players and athletes in other sports, favorable executive function performance was also found in soccer players. Specifically, soccer players outperformed track and field athletes in the tests of attention and executive control (Rahimi et al., 2022). Also, superior executive functions of working memory and cognitive flexibility were reported by comparing soccer players with boxers and shooters (Yongtawee et al., 2022).

The cross-sectional studies implied a positive role of soccer play in cognitive promotion, but the research design is inadequate to reach a causal effect. The experimental design, including comparisons between groups (soccer intervention vs. control) over time (pre-test vs. post-test), is effective in investigating a causal relationship between soccer play and cognitive performance. In a 6-month football intervention program, children (age: 8–9 years) attending soccer sessions indicated greater gains than their sedentary counterparts in attention, working memory, planning, and inhibition (Alesi et al., 2015, 2016). Further evidence was provided by a study which investigated acute effects of a single session on cognitive performance. A 60-min soccer training indued greater improvement than sedentary controls in visual attention, working memory, and long-term memory immediately after the session (Magistro et al., 2023). Consistent findings were identified in a 40-minute soccer practice (Wen et al., 2021). Two cognitive assessments, Go/No-Go test and 2-back test, were used as measures of inhibitory control and working memory, respectively. Significant interaction effects between group (soccer group vs sedentary group) and time (pre-test vs. post-test) were identified in both assessments, indicating larger improvement in the cognitive functions associated with the soccer session.

The enhanced cognitive performance after soccer training implies underlying adaptations at the neural level. Lind et al. (2019) implemented a 20-min small-sided soccer game session for the intervention group compared with the sedentary control group. In addition to the flanker task as a measure of inhibitory control, event-related potential (ERP) recorded cortical activities by P300 latency and amplitude. The improved cognitive performance in the soccer group was associated with larger increases in P300 amplitude at Fz in both congruent and incongruent trials, suggesting that adaptations in neural functioning contributed to the enhanced cognitive performance. Another study applied diffusion tensor imaging (DTI) to examine the influence of soccer juggling practice on white matter integrity (Shi et al., 2021). Compared with the control group which maintained routine life activities, the soccer group indicated a significant improvement in working memory as well as increased functional anisotropy (FA) in the genu of corpus callosum and the right anterior corona radiata. The research findings substantiated a positive relationship of the structural adaptations in the brain to the working memory performance after 10 weeks of soccer juggling practice.

## Integrating cognitive elements into basic drill practice

School settings provide children and adolescents with the opportunity to regularly participate in soccer training. However, feasible approaches need to be developed in consideration of the students' skill level. Although small-sided game has been widely applied in soccer training (Riboli et al., 2023), organizing soccer games for student novices can be challenging due to the incompetence of ball control as well as the lack of fundamental knowledge on rules and tactics. Accordingly, considerable efforts and time have to be allocated to improve basic skills such as passing and dribbling, which highlight the necessity of integrating cognitive elements into the basic drills. Cognitive-motor training (CMT) establishes an integrative training model. In a 10-week protocol for young players at a soccer academy, six cognitive-motor drills were designed with particular emphasis on executive functions, attention, and problem solving (Casella et al., 2022). By the end of the training program, players in the CMT group outperformed their counterparts in the control group in planning and visual search abilities, indicating that the integrative training is more effective than motor training alone (Casella et al., 2022).

**Figure 1:**
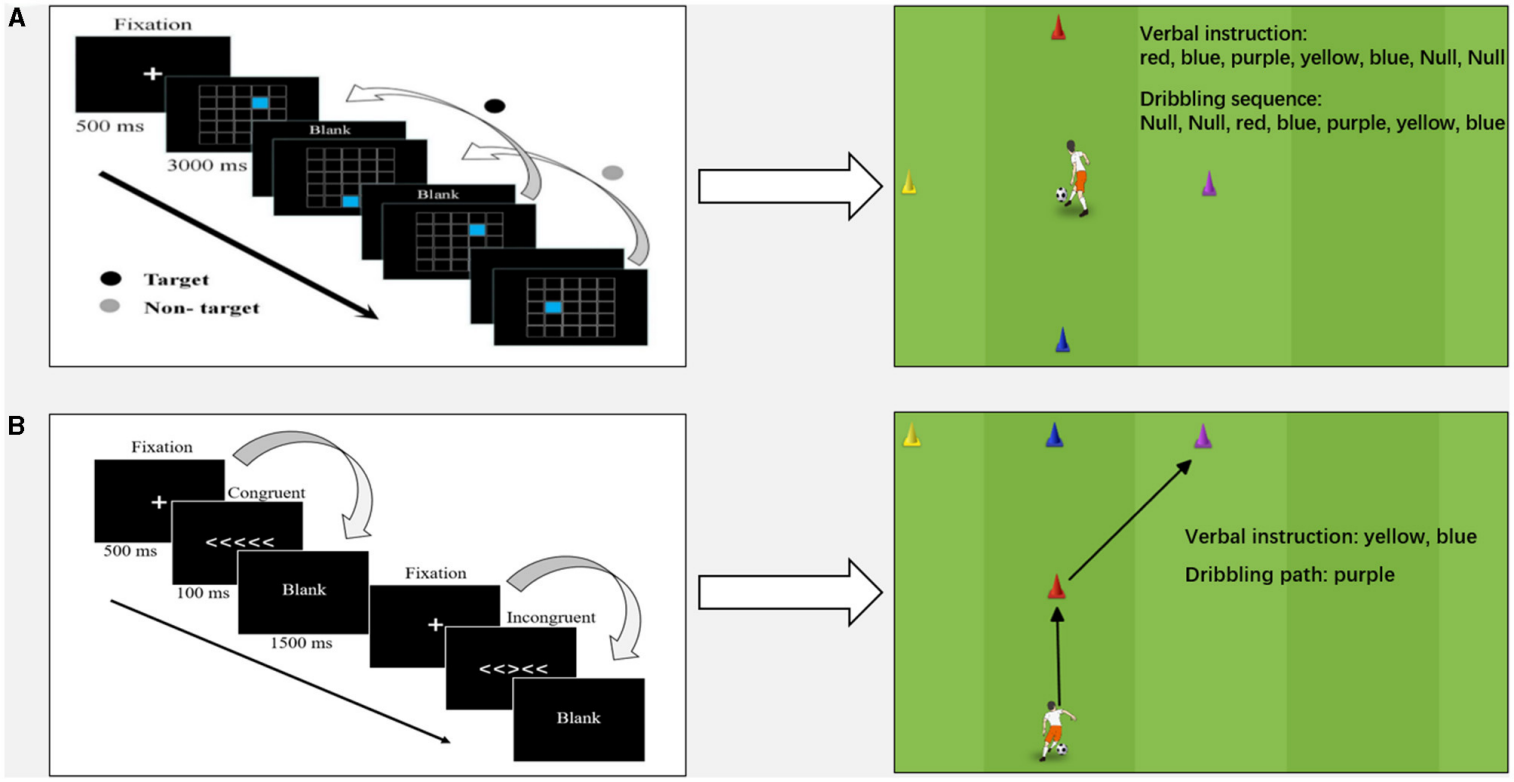
Illustration of the dribbling drills designed upon **(A)** the spatial 2-back task and **(B)** the flanker task. “Null” denotes as no movement or verbal instruction.

In line with CMT, integrative drills might be designed upon the commonly used cognitive tests. A typical paradigm for working memory test is the spatial 2-back task (Drollette et al., 2012). As shown in the left column of Figure 1A, a white plus symbol appears in the middle of the screen for 500 ms to raise participant's attention to the beginning of the test. The computer screen then displays 25 small squares. One of the squares is randomly highlighted in blue for 3000 ms, followed by a short black screen response interval before the next trial. Participants need to decide, as fast as they could, whether the position of a given stimulus matches the position of blue square presented in the previous two trials. Based on the procedures of the cognitive test, a training protocol that stimulates cognitive functions might be developed. The right column of Figure 1A displays the practice settings. The player begins from the center of an area which is marked by four cones in different colors. A PE teacher calls a series of colors (e.g., red-blue-purple-yellow-blue). Player does not move until the third color (purple) is called. In the meantime, the player dribbles toward the cone of the color called first (red). Then the player drives the ball to the second color (blue) while the teacher calls the fourth color of yellow. The player needs to notice the verbal stimulus while dribbling in the 2-back order. In this practice, cognitive activities can be stimulated as the player tries to keep the ball under control, search for the target, remember the order of colors, and plan the dribbling route.

Another example accounts for inhibitory control which is commonly assessed by the flanker task. The conventional computer-based test begins with a white plus symbol in the middle of the screen for 500 ms. Then five arrows are presented in congruent (all arrows pointing toward the same direction) or incongruent conditions (the central arrow pointing toward an opposite direction to the others). Participants need to make a quick response according to the direction of the central arrow (Eriksen and Eriksen, 1974). A dribbling practice that demands inhibitory control was displayed in Figure 1B. The player dribbles toward the red cone at which a color (e.g., blue) is called. The player needs to avoid the announced cone (e.g., the blue cone), but dribbling toward the unannounced one (e.g., yellow or purple cone). The practice can be more challenging if the teacher calls two colors (e.g., blue and yellow) at once, which leaves only one correct option (e.g., the purple cone) for the player to choose. In this case, the color called upon is a distractor that the player needs to inhibit in decision making and action.

## Discussion

Practicing basic skills accounts for a considerable amount of time in the regular training for student novices, thus integrating cognitive elements into basic drills (i.e., dribbling and passing) is essential in training design. By taking 2-back task and flanker test as the models of practice design, two dribbling drills were proposed as examples of integrating working memory and inhibitory control into practice. It is worth noticing two recent studies that established cognitive diagnostic tools in a soccer-specific setting (Musculus et al., 2022; Knöbel and Lautenbach, 2023). Participants made soccer-specific responses in the tasks of inhibition, cognitive flexibility, and working memory. Evidence proved validity and reliability of the assessment tools. Subsequent studies may work on combining integrative training with the soccer-specific cognitive instruments to establish a holistic training-assessment system to enhance cognitive promotion for soccer players.

The proposed integrative drills are suitable to after-school programs for student novices with particular interests in soccer. On the one hand, novices need to spend time learning and refining basic skills. Compared with conventional practice in which skills are repeated in a closed, static setting, integrative training in an interactive task stimulates cognitive activities during skill practice. On the other hand, the drills allow flexible arrangement in practice. The above examples described individual training. Also, a small group of players can practice in turns under a PE teacher's instruction.

Despite the promising effects of integrative soccer training, critical arguments should be raised about whether the enhanced cognitive performance is soccer specific. Existing studies compared soccer players with sedentary controls. The evidence is still inadequate to know whether playing soccer could result in superior improvement over other physical activities. Therefore, study design should be refined to provide further insights into effectiveness of soccer play on cognitive promotion. In terms of practical implementation, skill level is an important consideration. Skillful performance implies faster dribbling movement and higher accuracy in decision making. Due to speed-accuracy tradeoff, novices may slow down dribbling for accurate response. The prolonged movement times could reduce the frequency of stimuli and thus the effectiveness. PE teachers should find a balance between a player's skill level and task difficulty to optimize effects of the integrative training.

## Author contributions

FM: Methodology, Writing – original draft. ZL: Funding acquisition, Validation, Writing – review & editing. CQ: Software, Visualization, Writing – review & editing. QF: Conceptualization, Supervision, Validation, Writing – review & editing.
